# Retraction: Investigating new bilosomes for *ex vivo* skin deposition, *in vitro* characterization, and transdermal delivery of nimodipine

**DOI:** 10.1039/d6na90008a

**Published:** 2026-01-26

**Authors:** Ananda Kumar Chettupalli, Sarad Pawar Naik Bukke, Godswill James Udom, Tenpattinam Shanmugam Saraswathi, Shaik Abdul Rahaman, Sachchida Nand Rai, Marati Kavitha, Narender Boggula, Narayana Goruntla, Tadele Mekuriya Yadesa, Hope Onohuean

**Affiliations:** a Department of Pharmaceutical Sciences, School of Pharmacy, Galgotias University Greater Noida Uttar Pradesh-203201 India; b Department of Pharmaceutics and Pharmaceutical Technology, Kampala International University Western Campus, P.O.Box 71 Ishaka-Bushenyi Uganda; c Department of Pharmacology and Toxicology, Kampala International University Western Campus, P.O.Box 71 Ishaka-Bushenyi Uganda; d Department of Pharmaceutics, SRM College of Pharmacy, SRM Institute of Science and Technology Kattankulathur Tamilnadu-603203 India; e Centre of Experimental Medicine and Surgery (CEMS), Institute of Medical Sciences (IMS), Banaras Hindu University (BHU) Varanasi-221005 Uttar Pradesh India; f Department of Pharmacology, Raja Bahadur Venkata Rama Reddy (RBVRR) Women’s College of Pharmacy Barkatpura Hyderabad Telangana India; g Department of Pharmaceutical Chemistry, CMR College of Pharmacy Kandlakoya, Medchal Hyderabad Telangana 501 401 India; h Department of Clinical Pharmacy and Pharmacy Practice, Kampala International University Western Campus, P.O. Box 71 Ishaka-Bushenyi Uganda; i Biopharmaceutic Unit, Department of Pharmacology and Toxicology, Kampala International University Western Campus, P.O. Box 71 Ishaka-Bushenyi Uganda

## Abstract

Retraction of ‘Investigating new bilosomes for *ex vivo* skin deposition, *in vitro* characterization, and transdermal delivery of nimodipine’ by Ananda Kumar Chettupalli *et al.*, *Nanoscale Adv.*, 2024, Accepted Manuscript, https://doi.org/10.1039/d4na00510d.

The Royal Society of Chemistry hereby wholly retracts this *Nanoscale Advances* article due to concerns with the reliability of the data.

Fig. 11a of this article was first published in ref. 1 and reported as another compound.

Fig. 14 of this article was first published in ref. 2 and reported as another material.

In addition, following the publication of this article, a Royal Society of Chemistry journal received a submission with several shared co-authors that contained a piece of overlapping data with this published article.

We consulted an independent expert who asked for additional data from the TEM and histopathological experiments. The authors were not able to provide any additional data from their experiments.

Given the significance of these concerns, the Editor has lost confidence that the findings presented in this paper are reliable.

All authors were informed of the decision to retract this article. Ananda Kumar Chettupalli and Sarad Pawar Naik Bukke disagreed with the decision. Sachchida Nand Rai acknowledged the decision but did not agree nor disagree, and the other authors did not respond to any correspondence.

Signed: Jeremy Allen, Executive Editor, *Nanoscale Advances*

Date: 16th January 2026
